# Immunotherapy resistance in MASLD-related hepatocellular carcinoma: special immune microenvironment and gut microbiota

**DOI:** 10.7150/ijbs.117394

**Published:** 2025-09-03

**Authors:** Jie Jin, Kun Cheng, Mingrui Chen, Huifang Liang, Wanguang Zhang

**Affiliations:** 1 Hepatic Surgery Centre, Tongji Hospital, Tongji Medical College, Huazhong University of Science and Technology, Wuhan, Hubei 430030, People's Republic of China.; 2 Hubei Key Laboratory of Hepato-Pancreato-Biliary Diseases, Wuhan, Hubei 430030, People's Republic of China.; 3 Clinical Medicine Research Centre for Hepatic Surgery of Hubei Province, Wuhan, Hubei 430030, People's Republic of China.; 4 Key Laboratory of Organ Transplantation, Ministry of Education; NHC Key Laboratory of Organ Transplantation; Key Laboratory of Organ Transplantation, Chinese Academy of Medical Sciences, People's Republic of China.

**Keywords:** metabolic dysfunction-associated steatotic liver disease, hepatocellular carcinoma, immune microenvironment, gut microbiota, immunotherapy

## Abstract

Obesity represents a major global public health challenge. Consequently, metabolic dysfunction-associated steatotic liver disease (MASLD) has become the primary driver of chronic liver disease globally and is currently the most rapidly accelerating factor contributing to hepatocellular carcinoma (HCC). However, current evidence indicates that immunotherapy, a cornerstone of HCC management, yields suboptimal results specifically in MASLD-related HCC (MASLD-HCC) cases. Various immune components constitute a special immune microenvironment in MASLD-HCC, including heterogeneous myeloid cells, lymphocytes and platelets. Furthermore, disruptions in the intestinal barrier, along with the ectopic presence of intestinal flora and metabolites, also influence the immune microenvironment in MASLD-HCC. Elucidating immune cells functions and their interplay with gut microbiota is critical to deciphering MASLD progression to carcinogenesis and immunotherapy resistance. This review synthesizes current insights into the immune microenvironment and gut microbiome in MASLD-HCC, identifies factors influencing the efficacy of immunotherapy, and summarizes potential therapeutic targets to provide detailed guidance for developing effective immunotherapy strategies for MASLD-HCC.

## Introduction

Metabolic dysfunction-associated steatotic liver disease (MASLD), the successor diagnosis to non-alcoholic fatty liver disease (NAFLD), currently impacts an estimated 25% of people globally. Fueled by growing epidemics of obesity and metabolic syndrome, this condition has become the primary cause of chronic liver disease worldwide 1-3. Although the term NAFLD, first introduced by Ludwig et al. in 1980, has been used for nearly half a century, it was formally replaced by MASLD in June 2023. This change, based on a consensus document jointly released by international liver societies, addresses concerns regarding the term's ambiguous exclusionary diagnostic criteria and its stigmatizing nature 4. MASLD encompasses both a relatively benign, non-progressive phenotype characterized by ≥5% hepatic steatosis, and progressive metabolic dysfunction-associated steatohepatitis (MASH). Steatosis, lobular inflammation, hepatocellular ballooning, and fibrosis represent the defining histological features of MASH, a condition with potential for progression to cirrhosis and hepatocellular carcinoma (HCC) 5, 6. HCC comprises the majority of primary liver cancers, stands as the sixth most prevalent cancer diagnosis, and is the third most common cause of cancer-related death 7. It is well known that hepatitis B virus (HBV) and hepatitis C virus (HCV) infections act as the most important drivers of primary liver cancer 8, 9. The role of viral hepatitis in HCC etiology has declined ascribed to the greater vaccination coverage and antiviral medications, which can suppress viral effectivity. Accumulating epidemiological evidence reveals that MASLD is the fastest-growing etiological driver of HCC incidence globally 10, 11. The underlying mechanisms of MASLD-related HCC (MASLD-HCC) include excessive lipid accumulation and lipid-induced hepatic insulin resistance (IR), liver cell damage caused by dysregulated metabolism, unique host genetic variants, gut microbiota and their metabolic products, and chronic inflammation-induced immune response 12, 13.

Contemporary HCC management encompasses surgical options (resection, transplantation), ablation, transarterial approaches, radiotherapy, and systemic therapies consisting of tyrosine kinase inhibitors (TKIs) and immune checkpoint inhibitors (ICIs) 7, 14, 15. Due to the confounding effects of subcutaneous adipose tissue and hepatic steatosis on ultrasonographic accuracy, coupled with the recognition that not all MASLD patients progressing to HCC traverse a cirrhotic pathway, MASLD-HCC is frequently diagnosed at advanced stages 16-18. Mounting clinical evidence reveals an etiological stratification in HCC responsiveness to immunotherapy. Two meta-analyses, respectively including 8 trials with 3739 patients and 3 trials with 1656 patients, revealed significantly greater efficacy of ICIs in viral-related HCC compared to nonviral HCC. In contrast, the efficacy of TKIs showed no etiological dependence 19, 20. Crucially, MASLD-HCC exhibits profound immunotherapy resistance, evidenced by two independent cohorts reporting significantly reduced median overall survival compared to other etiologies. This consistent survival disadvantage delineates a distinct resistance phenotype inherent to metabolic dysfunction-driven hepatocarcinogenesis 19.

The efficacy of ICIs, including anti-programmed death receptor-1 (PD-1), anti-programmed death-ligand 1 (PD-L1), and anti-cytotoxic T-lymphocyte antigen 4 (CTLA4) mAbs, is largely determined by the composition and state of the tumor immune microenvironment. MASLD-HCC exhibits a unique immunometabolic microenvironment. Lipid-laden macrophages promote hepatocyte lipid accumulation, while deficiencies in nuclear receptor coactivator 5 (NCOA5) or neuregulin 4 (NRG4) drive macrophages polarization towards a tumor-associated macrophages (TAMs)-like phenotype, accelerating MASLD-driven hepatocarcinogenesis 21-23. Accumulated polyunsaturated fatty acids (PUFAs) foster neutrophil extracellular traps (NETs) generation that shifts naïve CD4+ T cells differentiation toward regulatory T cells (Tregs) 24. Concurrently, CD8+ T cells display functional impairment, increased exhaustion, and reduced motility 19, 25. The lipid-enriched milieu also depletes CD4+ T cells and induces linoleic acid-mediated oxidative damage, further promoting tumorigenesis 26. Immunosuppressive IgA+ B cells accumulate in MASLD-HCC, impairing antitumor immunity by inhibiting cytotoxic CD8+ T cells 27. Diverse immune cells coordinate an immunosuppressive milieu conducive to tumorigenesis, significantly exacerbating MASLD-HCC transition dynamics, though their precise individual roles warrant further elucidation. Pathogenic alterations in these bidirectional signaling pathways trigger a sequence of pathological events that culminate in metabolic diseases, with MASLD being a prominent example 28. Gut dysbiosis contributes to MASLD pathogenesis by compromising intestinal barrier integrity, thereby facilitating the translocation of microbiota-derived factors and microbial-associated molecular patterns (MAMPs) to the liver. Engagement of hepatic pattern recognition receptors (PRRs), notably Toll-like receptors (TLRs), by these molecules initiate potent pro-inflammatory pathways, driving increased hepatic inflammation and fibrogenesis 12. This review also delineates how gut microbiota-derived signals modulate the hepatic immune landscape, offering novel perspectives on immunological perturbations in MASLD progression.

Elucidating the changes in the immune system during MASLD-HCC progression can provide crucial insights into the potential mechanisms behind the reduced effectiveness of immunotherapy in MASLD-HCC patients. In this review, we discuss the innate and adaptive immune responses, alongside gut microbiota and metabolite-mediated immunological shifts in MASLD-HCC pathogenesis. Accumulating a deeper understanding of these immune mechanisms may provide new insights into MASLD-HCC development and help improve the efficacy of prevention and immunotherapy strategies for MASLD-HCC.

## Myeloid Cell and Regulation of the MASLD-HCC Immune Microenvironment

### Macrophages

**Figure 1:**
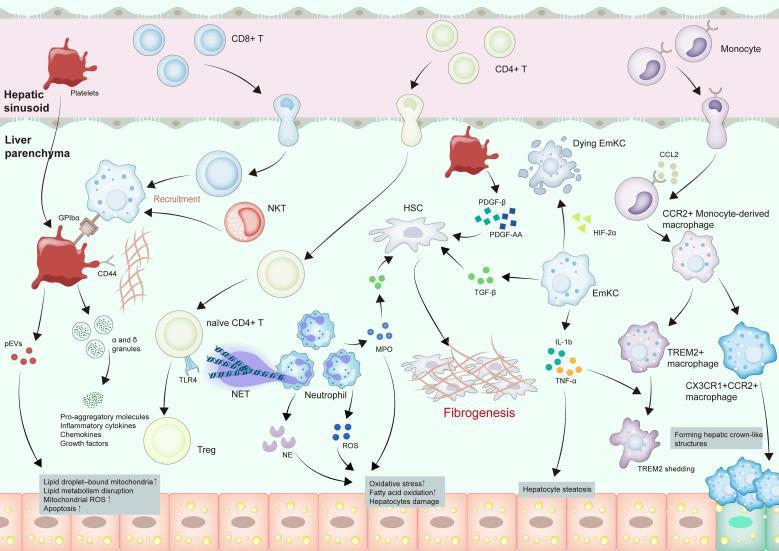
**Innate immune modulation and platelets in MASLD-HCC pathogenesis.** CCL2 secreted by steatotic hepatocytes recruits monocyte-derived macrophages, which then differentiate into anti-inflammatory TREM2+ macrophages and proinflammatory CX3CR1+CCR2+ macrophages. CX3CR1+CCR2+ macrophages participate in the formation of hepatic crown-like structures. EmKCs are also activated, which secrete IL-1β and TNF-α to induce TERM2 shedding in TREM2+ macrophages and aggravate hepatocyte steatosis. Neutrophils produce MPO, NE, and ROS, increasing oxidative stress and hepatocyte damage. NETs form and recruit naïve CD4+ T cells, driving TLR4-dependent Tregs differentiation. Platelets are increased and activated during the progression. pEVs containing impaired mitochondria are transferred to hepatocytes, causing excess LD buildup, heightened mitochondrial ROS, and apoptosis. The α and δ granules secreted by platelets release particles laden with pro-aggregatory molecules, inflammatory cytokines, chemokines, and growth factors, potentiating inflammatory responses. Platelets recruit CD8+ T cells and NKT cells by hyaluronan-CD44 binding with Kupffer cells in a platelet membrane GPIbα-dependent manner. HSCs are activated by TGF-β from macrophages, MPO from neutrophils, and PDGF-β or PDGF-AA from platelets, finally causing fibrogenesis. Abbreviations: CCL, C-C motif chemokine ligand; CX3CR1, C-X3-C motif chemokine receptor 1; EmKC, Embryonic Kupffer cell; GPIbα, glycoprotein Ibα; HCC, hepatocellular carcinoma; HSCs, hepatic stellate cells; IL-1β, interleukin 1 beta; pEVs, platelet-derived extracellular vesicles; PDGF, platelet-derived growth factor; LD, lipid droplet; MPO, myeloperoxidase; MASH, metabolic dysfunction associated steatohepatitis; MASLD-HCC, metabolic dysfunction associated steatotic liver disease-related hepatocellular carcinoma; NE, neutrophil elastase; ROS, reactive oxygen species; NETs, neutrophil extracellular traps; NKT, natural killer T cells; TGF-β, transforming growth factor beta; TLR4, toll-like receptor 4; TNF-α, tumor necrosis factor alpha; Tregs, regulatory T cells; TREM2, triggering receptor expressed on myeloid cells 2.

Macrophages, present as abundant resident cells throughout the body's organs, are integral to tissue development and homeostasis while potentially playing a role in diverse ailments pathogenesis 29, 30, including MASLD **(**Figure 1). Macrophages can be categorized by origin into embryo-derived Kupffer cells (EmKC) and bone marrow/monocyte-derived macrophages 30, 31. EmKCs form the predominant resident macrophage subset. Their functional repertoire, encompassing the secretion of anti-inflammatory mediators and proficient phagocytosis of particulates arriving through the portal circulation, is essential for sustaining liver immune equilibrium 32, 33. Bone marrow/monocyte-derived macrophages, however, infiltrate liver tissue during liver injury or inflammation, exhibiting proinflammatory features 34, 35. Macrophages can also be divided into inflammatory, lipid- and scar-associated, and restorative macrophages based on their different functions 30, 36, 37.

The occurrence and progression of MASLD are inseparable from significant macrophages involvement 32, 38. During hepatic steatosis onset, the deposition of excess fatty acids places the liver in a state of stress, cholesterol, chemokine C-C motif chemokine ligand 2 (CCL2) and C-X-C motif chemokine ligand 10 (CXCL10) secreted by steatotic hepatocytes activate macrophages 39, 40. Activated Kupffer cells can inhibit hepatocyte lipid metabolism through paracrine release of interleukin 1 beta (IL-1β) and tumor necrosis factor (TNF) α, ultimately encouraging hepatocyte steatosis 41. In the pro-inflammatory hepatic milieu, EmKCs exhibit compromised self-renewal potential 42, 43. The recruitment of C-C motif chemokine receptor 2 (CCR2) + monocyte-derived macrophages into the liver suppresses intrahepatic hepatic triglyceride (TG) retention and drives disease evolution toward steatohepatitis 43. This process is further amplified by paracrine signaling between macrophages and hepatic stellate cells (HSCs), wherein transforming growth factor beta (TGF-β) acts as a key effector to stimulate pro-fibrotic HSC activation, thereby facilitating MASH development 44, 45.

In MASH progression, lipid-associated macrophages, a monocyte-derived subset, comprise two phenotypes: transitional CX3CR1+CCR2+ macrophages and classic triggering receptor expressed on myeloid cells 2 (TREM2) + macrophages 46. TREM2+ macrophages exhibit anti-inflammatory effects by modulating lipid uptake, thereby mitigating MASH progression, while CX3CR1+CCR2+ macrophages contribute to hepatic crown-like structures formed by macrophages encircling lipid-laden hepatocytes 46. Hepatic crown-like structures serve as histological markers of advanced disease, with their density closely tracking the severity of liver fibrosis 47. In MASH, IL-1β and TNF-α induce TREM2 shedding via a disintegrin and metalloproteinase 17 (ADAM17)-mediated proteolytic cleavage, leading to abnormal accumulation of dying hepatocytes, which exacerbates proinflammatory cytokine production and drives disease progression 48. Hypoxia-inducible factor (HIF) has emerged as a pivotal regulator of immune function and inflammatory pathways 49. Within macrophages in MASH mouse models, elevated HIF-1α levels impair autophagic flux while promoting IL-1β secretion. Concurrently, HIF-1α-driven NF-κB activation upregulates monocyte chemoattractant protein-1 (MCP-1), with both cytokines exacerbating hepatic steatosis and inflammatory responses 50. HIF-2α exerts cell type-specific effects in liver macrophages: it compromises EmKC homeostasis by aggravating lysosomal stress, resulting in diminished proliferation and phagocytosis. In contrast, bone marrow/monocyte-derived macrophages undergo HIF-2α-driven pro-inflammatory polarization via mitochondrial ROS amplification and coordinated upregulation of inflammasome-related genes 51.

The role played by macrophages in MASLD-HCC is incompletely understood. Some studies have discovered that myeloid differentiation primary response 88 (MyD88) in myoblasts enhances MASLD-HCC development by promoting M2 macrophage polarization 52. NCOA5 deficiency in macrophages was also identified as a key factor in the transition 22, 53. Previous studies identified NRG4 as a regulatory checkpoint suppressing tumor-permissive liver microenvironments. Loss of NRG4 promotes macrophages with TAM-like properties and drives cytotoxic CD8+ T cells exhaustion in MASLD-HCC 21. A study demonstrated a significant upregulation of the YT521-B homology (YTH) m6A RNA-binding protein 1 (YTHDF1) in MASLD-HCC compared to peri-tumor regions. Upregulated YTHDF1 promotes MASLD-associated carcinogenesis through EZH2-IL6 pathway stimulation. This signaling cascade recruits and activates myeloid-derived suppressor cells (MDSCs), ultimately suppressing CD8+ T cells cytotoxicity 54. Besides, former studies indicated that tumor-activated monocytes exhibit robust PD-L1 surface expression, which potently suppresses T cells function and accelerates HCC progression 55, 56.

During MASLD progression to HCC, distinct macrophage subsets drive lipid accumulation and inflammation through cytokine secretion. Critically, macrophage dysfunction—manifested by MyD88-dependent M2 polarization, NCOA5 deficiency, NRG4 loss, and YTHDF1-EZH2-IL6-mediated MDSCs recruitment—establishes a profoundly immunosuppressive microenvironment. This axis may play a pivotal role in both MASLD-HCC development and resistance to immunotherapy.

### Neutrophils

Circulating neutrophils, being the most numerous white blood cells, execute frontline protective functions within the innate immune framework 57. However, abnormally activated neutrophils are associated with certain inflammation-related diseases 58, 59. Studies have shown that neutrophils infiltration is frequently noted in MASLD patients and correlates with disease progression 60, 61. Neutrophils drive MASLD-HCC pathogenesis through ROS generation, protease secretion, and NETs formation 62, 63 (Figure 1).

In MASLD liver, upregulated CXCL1 expression recruits neutrophils, leading to ROS production, which promotes the transition from steatosis to steatohepatitis by inducing oxidative stress and activating related signaling pathways 64, 65. IL-8 also contributes to the recruitment of neutrophils to the liver and promotes MASH by overexpressing CXCL1 and inducing mitochondrial oxidative stress 66. IL-22, on the other hand, can upregulate hepatic antioxidant enzymes, metallothionein (MT) 1 and MT2, to impede neutrophils recruitment, thereby alleviating MASH development 67.

The proteolytic enzymes released by neutrophils—notably myeloperoxidase (MPO), neutrophil elastase (NE), and human neutrophil peptides (HNPs) may substantially contribute to MASLD development. MASH is characterized by increased MPO levels compared with simple steatosis 67. In the MASLD mouse model, MPO catalyzes HOCl formation from H₂O₂, which damages hepatocytes and upregulates TGF-β, activating HSCs to drive fibrosis 68. NE has multiple roles, including pro-inflammatory and pro-cancer effects 69, 70. It has been demonstrated that NE promotes inflammation and insulin resistance by modulating the AMPK pathway and fatty acid oxidation 71, 72. HNPs also promote the transition of MASH to fibrosis by stimulating HSCs proliferation 73.

NETs represent extracellular chromatin networks where unpacked DNA scaffolds embed neutrophil-derived granular enzymes and cytosolic proteins 74, 75. Studies have indicated that the accumulation of PUFAs drives NETs formation in MASH progression 76. In MASLD-HCC, NETs regulate the mitochondrial oxidative phosphorylation (OXPHOS) of naïve CD4+ T cells, driving their differentiation into Tregs in a TLR4-dependent manner. This process establishes an immunosuppressive microenvironment, promoting HCC development in MASH 24, 60.

Intercellular crosstalk critically modulates MASH progression. Within the MASLD-HCC tumor microenvironment (TME), CXCR2-expressing neutrophils demonstrate significant spatial enrichment, secreting diverse protumor mediators 77. This phenotypic profile confers upon them the capacity to orchestrate a cascade of immunomodulatory events, including MDSC activation, the inhibition of dendritic cells maturation and function, and the promotion of tumor progression 77. Lipocalin (LCN)-2 secreted by neutrophils also upregulates CXCR2 to facilitate the recruitment and proliferation of pro-inflammatory macrophages via NF-κB signaling 78. Besides, NETs may also attract monocyte-derived macrophages to infiltrate the liver by releasing certain signaling molecules or altering the local microenvironment 60. Additionally, microRNA-223 can be taken up by hepatocytes through the binding of low-density lipoprotein receptor (LDLR) and apolipoprotein E (APOE), thereby inhibiting MASH progression 79.

Taken together, neutrophils drive MASLD progression, fibrogenesis, and HCC pathogenesis by recruiting CXCR2+ neutrophils, producing ROS, proteases, LCN-2, and microRNA-223, and forming NETs.

### Dendritic cells

Positioned at the innate-adaptive interface, dendritic cells (DCs) coordinate initial defense reactions while instigating antigen-driven lymphocyte activation 80, 81. DCs differentiate into three principal subsets based on ontogeny: conventional DCs (cDCs), plasmacytoid DCs (pDCs), and Langerhans cells (LCs) 80, 82. cDCs comprise two principal subtypes: conventional type I dendritic cells (cDC1) and conventional type II dendritic cells (cDC2) 83.

Previous studies have reported a decline in CD8+ pDCs and CD11c+CD8+ α-DCs during MASLD, concomitant with an increase in CD11b+CD8- pDCs 84. CD103+ cDC1s and CD11b+ cDC2s also accumulate in the MASLD process 85, 86. CD130+ DCs were discovered to serve as hepatoprotective agents in MASLD by regulating the immune response, limiting inflammatory cell activation, and potentially removing cell debris, thereby mitigating steatosis in MASLD 85, 87. In contrast, chemokine X-C receptor 1 (XCR1) + cDC1s accumulate in MASH-affected livers across species, with their density positively correlating with histological severity 88, 89. Mechanistically, liver pathology results from an excess of cDC1s, generated by enhanced proliferation of their bone marrow precursors. These amplified cDC1 populations drive inflammation by activating and reprogramming pro-inflammatory T cells 88. However, some studies have yielded different conclusions. *Batf3*-deficient mice, which lack cDC1s, exhibit significant inhibition of the transition from steatosis to steatohepatitis 90. The observed discrepancies may stem from the fact that the deletion of Batf3 may also impacts other immune cells. Furthermore, preclinical investigations indicated that co-blockade of PD-1 and CXCR2 significantly augments the XCR1+ cDC1s population, which promotes CD8+ T cells recruitment, thereby enhancing the therapeutic efficacy of the combination regimen 77.

DCs exhibit dichotomous roles in MASLD-HCC. Protective subsets like CD130+ DCs mitigate steatosis by regulating inflammation and clearing debris. Conversely, pathogenic XCR1+ cDC1s orchestrate pathology by activating pro-inflammatory T cells. Therapeutically, augmenting specific DC subsets (e.g., XCR1+ cDCs via anti-PD-1/CXCR2 inhibition) represents a therapeutic strategy to amplify CD8+ T cells infiltration and potentiate treatment efficacy.

## Lymphocyte-Mediated Immune Response in MASLD-HCC

### CD8+ T cells

The pathogen-clearing function of CD8+ T cells—mediating long-lasting protective immunity and homeostatic balance—demonstrates a significant association with improved patient survival metrics in HCC patients 91-93. Depleting CD8+ T cells or administering anti-CD8α treatment promotes MASLD-HCC development 27, 77. ICIs-based immunotherapy is also implemented based on their characteristics. However, some studies have revealed that CD8+ T cells in MASLD-HCC fail to exert anti-tumor effects and may even promote MASLD-HCC progression 19, 94. Many studies have dedicated efforts to elucidating this intriguing but paradoxical phenomenon.

Quantitatively, despite an elevated systemic frequency, CD8+ T cells often exhibit impaired tumor infiltration, compromising their anti-tumor efficacy. During MASH progression, CD8+ T cells migrate to the liver via antigen presentation or cytokine signaling, increasing their overall population 95-98. However, hepatic CD8+ T cells infiltration is primarily hindered by excessive collagen fiber deposition at the tumor margin 99. Recent advances in spatial transcriptomics have also revealed that immune cells are predominantly enriched in adjacent normal tissues but markedly diminished within tumor regions 100. This spatially marginal distribution pattern and diminished infiltration capacity of CD8+ T cells constrain their antitumor efficacy, resulting in immunotherapy being predominantly effective at tumor margins rather than within the tumor core, ultimately contributing to MASLD-HCC development and immunotherapy inefficiency.

Functionally, CD8+ T cells shift from naïve or effector states to dysfunctional or exhausted states, characterized by expanding intrahepatic CD8+PD-1+ T cells expressing genes linked to exhaustion, tissue residency, and impaired effector functions 19, 94, 96. Compared to healthy individuals, MASLD-HCC patients exhibit higher rates of catenin beta 1 (CTNNB1) mutations, which elevate tumor necrosis factor receptor superfamily 19 (TNFRSF19) levels and suppress the secretion of senescence-associated secretory phenotype (SASP)-like cytokines, such as IL-6 and CXCL8, fostering an immune-excluded 'cold' TME that exacerbates CD8+ T cells dysfunction 101, 102. Besides, overexpressed YTHDF1 has been implicated in suppressing cytotoxic CD8+ T cells function by enhancing IL-6 secretion 54. Cholesterol accumulation dysregulates CD8+ T cells cytotoxicity through suppressed granzyme B (GZMB) and interferon gamma (IFN-γ) secretion 103. Intratumoral CD8+ T cells in MASH-bearing mice additionally exhibit impaired motility—evidenced by reduced migration velocities and shortened displacement lengths—collectively diminishing antitumor capacity 25, 94. Altered hepatic lipid metabolism likely drives CD8+ T cells metabolic reprogramming in MASH pathogenesis. Supporting *in vitro* data reveal that MASH impairs tumor-infiltrating CD8+ T cells motility independently of chemokine signaling or adhesion molecule interactions. Metabolic profiling of CD8+ T cells derived from NASH mice reveals dysregulated glycolysis, fatty acid oxidation, and mitochondrial respiration, substantiating their functional impairment. This metabolic impairment is further evidenced by marked mitochondrial depolarization and diminished mitochondrial mass 25, 94, 96. Therefore, despite the increase in CD8+ T cells, their functionality is predominantly impaired, rendering them unable to exert anti-tumor effects, contributing to MASLD-HCC development and immunotherapy inefficiency.

**Figure 2:**
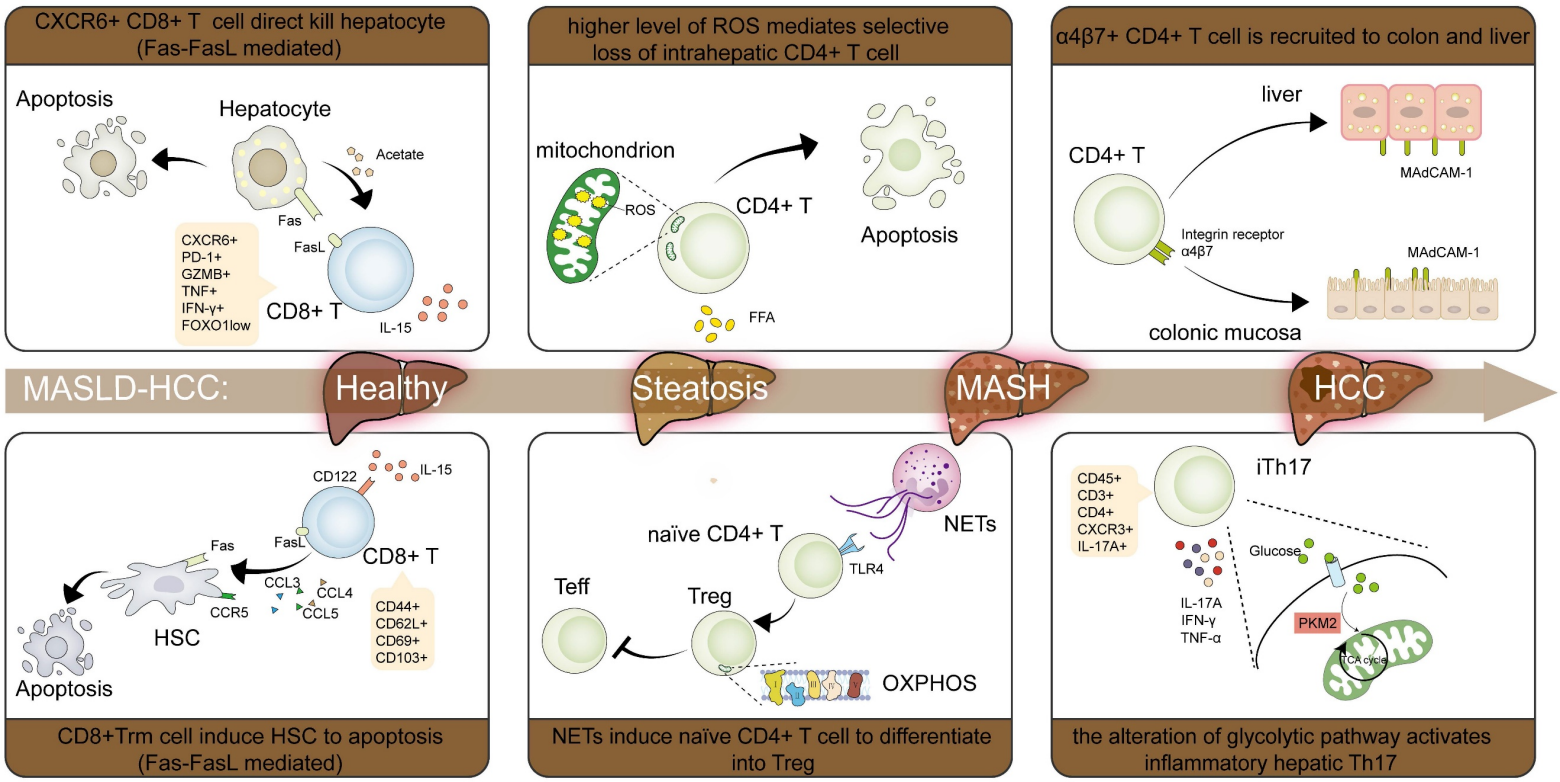
** Characteristic changes of T cells in MASLD-HCC pathogenesis.** In CD8+ T cells, IL-15 populated the CXCR6+PD-1+CD8+ T cells and CD8+ Trm cells, which cause HSCs apoptosis through Fas/FasL interaction respectively. Some CD8+ T cells also exhibit lower velocities and shorter displacement lengths, thus reducing their motility and antitumor effect. In CD4+ T cells, high levels of ROS make CD4+ T cells much more vulnerable to exposure to lipid metabolites, finally causing CD4+ T cells apoptosis. α4β7+ CD4 T cells are recruited by increased MAdCAM-1 in the liver and colon, which exacerbates inflammation and fibrosis. CXCR3+Th17 (ihTh17) is also increased to contribute to the development of MASH through activating the CXCR3-CXCL9/10 axis and reprogramming the metabolic and proinflammatory phenotype. The formation of NETs regulates the mitochondrial OXPHOS of naïve CD4+ T cells and drives their differentiation into Tregs in a TLR4-dependent manner. Abbreviations: CXCR, C-X-C motif chemokine receptor; CXC, C-X-C motif chemokine ligand; Fas, factor associated with suicide; FasL, Fas ligand; HCC, hepatocellular carcinoma; HSCs, hepatic stellate cells; IL-15, interleukin 15; MAdCAM-1, mucosal addressin cell adhesion molecule-1; MASH, metabolic dysfunction associated steatohepatitis; MASLD-HCC, metabolic dysfunction associated steatotic liver disease-related hepatocellular carcinoma; OXPHOS, oxidative phosphorylation; NETs, neutrophil extracellular traps; PD-1, programmed death receptor 1; ROS, reactive oxygen species; TLR4, Toll-like receptor 4; Tregs, regulatory T cells; Trm, tissue-resident memory T cells.

A unique CXCR6+CD8+ T cells subset that exerts distinct roles compared with other CD8+ T cells subsets has been identified. CXCR6+CD8+ T cells maintenance depends on CCR7+ DCs that express the cognate ligand CXCL16 and provide IL-15 cytokine signaling 104. These T cells, characterized by low Forkhead Box O1 (FOXO1) activity, are detected to accumulate in MASH mice fed a choline-deficient, high-fat diet (CD-HFD) or a western diet (WD). Mechanistically, IL-15-mediated FOXO1 suppression coupled with CXCR6 induction metabolically sensitizes CXCR6+CD8+ T cells. This reprogramming enables aberrant recognition of acetate/ATP signals, provoking auto-aggressive cytolysis through factor associated with suicide (Fas) / Fas ligand (FasL) interaction 105. Strikingly, CXCR6+CD8+ T cells exhibit heightened migratory velocity when activated by local tissue signals 94 (Figure 2).

Therefore, immunotherapy resistance in MASLD may arise from: reduced tumor CD8+ T cells infiltration, functionally exhausted T cells with impaired motility, and pathological accumulation of CXCR6+ or PD-1+ CD8+ T cells subset in the liver. Cell metabolism is widely recognized as a factor affecting T cells function and migration. Given that MASLD is characterized by metabolic dysregulation, alterations in the MASLD-HCC TME may induce metabolic disturbances in CD8+ T cells, potentially exacerbating hepatic damage and promoting MASLD-HCC.

### CD4+ T cells

As master regulators of adaptive immunity, CD4+ T cells represent a fundamental lymphocyte subpopulation. These cells segregate into two functional lineages: helper T cells (Th) and Tregs 106, 107. Th cells undergo further specialization into distinct subsets (Th1, Th2, Th17, Th22) defined by unique transcriptional programs and cytokine signatures 107, 108. CD4+ T cells subsets exert their respective effects, promoting or inhibiting disease progression, thus forming an immune regulatory network. Progression of MASH is accelerated through IFN-γ/TNF-α secretion from Th1 cells, mediating hepatocyte cytotoxicity and inflammation potentiation 109, 110. Th2 cells, by secreting IL-13, promote HSC activation, leading to liver fibrosis 111. Th17 cells accelerate MASH progression by secreting IL-17, which promotes hepatocellular injury and inflammatory responses 112, 113. IL-17 also promotes hepatic fibrosis by up-regulating TGF-βRII on HSCs surfaces, which enhances their response to TGF-β 114. In contrast, Th22 cells have a protective effect by secreting IL-22, attenuating hepatocytes injury and inflammatory response 115, 116.

In methionine-choline-deficient (MCD) or choline-deficient and amino acid-defined (CDAA) diet-induced MASH models, intrahepatic CD4+ T cells depletion occurs as lipid-rich microenvironments upregulate CPT expression. This amplifies mitochondrial biogenesis and reactive oxygen species generation, enhancing susceptibility to cytotoxic lipid metabolites like linoleic acid 26, 117. Reports indicate a decrease in total hepatic CD4+ T cells**,** yet some subpopulations increase in MASH. Expanded central and effector memory CD4+ T cells drive liver inflammation and fibrosis 118. Th17 cells are also demonstrated to be increased 119, 120. A significant study uncovered the presence of a unique pathogenic subpopulation of liver Th17 cells, inflammatory hepatic CXCR3+ Th17 (ihTh17), which is sufficient to contribute to MASLD development through activating the CXCR3-CXCL9/10 axis and reprogramming cells toward a metabolic and proinflammatory phenotype 121. Besides, enhanced hepatic and intestinal mucosal addressin cell adhesion molecule-1 (MAdCAM-1) expression in WD-fed mice facilitated α4β7+ CD4+ T cells recruitment, directly aggravating inflammatory responses and extracellular matrix deposition in the liver 122.

Tregs, a highly immunosuppressive subset of CD4+ T cells characterized by CD4+FOXP3+CD25+ expression, are essential for maintaining an immunosuppressive microenvironment 123. Emerging evidence reveals a biphasic role of Tregs in MASLD pathogenesis 124. During early steatosis, obesity and insulin resistance suppress Tregs differentiation and impair their functional capacity 125, 126. Concurrently, oxidative stress triggers Tregs apoptosis and activates the TNF-α signaling pathway, collectively driving progressive hepatic injury 127. As disease advances to MASH, substantial Tregs expansion occurs despite this initial suppression. NETs reprogram mitochondrial OXPHOS in naïve CD4+ T cells via TLR4 signaling, promoting their differentiation toward a regulatory phenotype over an effector phenotype 24. During chronic liver injury, these elevated Tregs produce amphiregulin, which engages epidermal growth factor receptor (EGFR) on HSCs. This interaction directly promotes hepatic fibrogenesis and concurrently stimulates HSCs to secrete IL-6. The resulting IL-6 contributes to glucose intolerance, thereby establishing a vicious cycle that further drives MASH progression 128. Gut dysbiosis represents a well-recognized pathological feature of MASLD. Microbial-derived short-chain fatty acids (SCFAs) amplify Tregs responses by enhancing IL-10-secreting Tregs abundance and expanding specialized effector Tregs populations 129. As a microbial membrane constituent, lipoteichoic acid (LTA) translocates from gut to liver parenchyma, directly driving senescence programming in HSCs with consequent SASP factor secretion. Critically, bioactivation of IL-33 occurs through chymotrypsin-like elastase family member 1 (CELA1)-mediated proteolytic cleavage of its full-length precursor. Senescent HSCs export this mature IL-33 via gasdermin D (GSDMD) amino-terminal domain-mediated pore formation. The liberated cytokine then engages ST2+ Tregs (where ST2 functions as the IL-33 receptor), driving obesity-promoted hepatocarcinogenesis 130.

Herein, the metabolic reprogramming in CD4+ T cells is evident in MASLD-HCC pathogenesis. These cells exhibit increased mitochondrial mass and elevated mitochondrial OXPHOS activity. Heightened mitochondrially derived ROS instigates oxidative stress-mediated depletion of intrahepatic CD4+ T cells, accelerating hepatocarcinogenesis. Besides, enhanced OXPHOS activity directs naïve CD4+ T cells commitment to the Treg lineage, sustaining an immunosuppressive microenvironment (Figure 2).

### B cells

**Figure 3:**
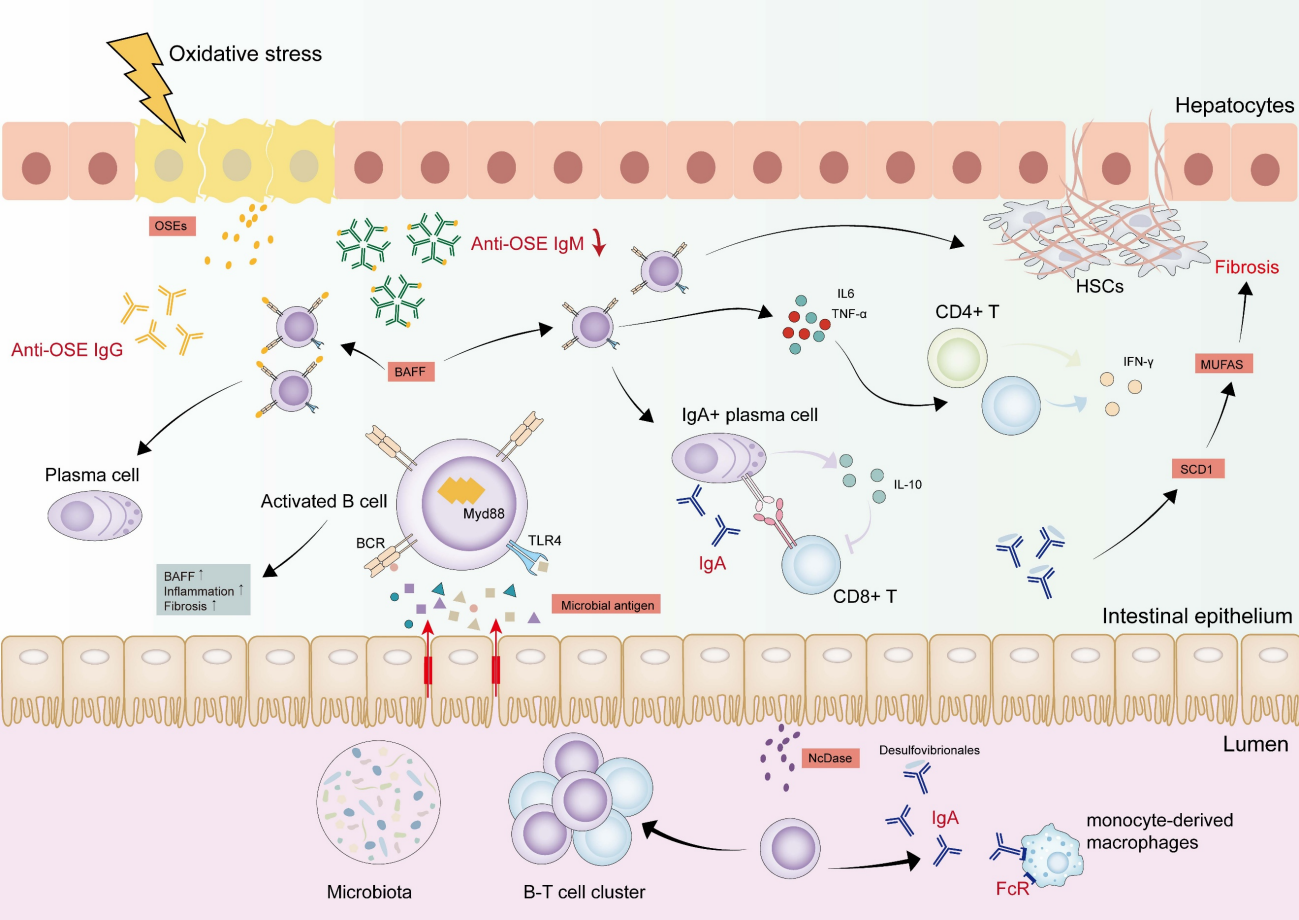
** Dynamic changes of B cells in MASLD-HCC pathogenesis**. B cells were activated by antigens derived from the gut and bacterial metabolites draining into the liver through MyD88-dependent or BCR signaling. The activated B cells are associated with increased inflammation and fibrosis, as well as BAFF, which forces B cell survival and maturation. IL-6 and TNF-α derived from intrahepatic B cells activate CD8+ T cells and CD4+ T cells, facilitating their secretion of IFN-γ. B cells also activate HSCs and promote fibrosis dependent on TNF signaling. IgA+ B cells are accumulated in the liver, which suppress CD8+ T cells by upregulating the expression of PD-L1 on the cell surface and producing the immunosuppressive cytokines. In the gut, IgA activates monocyte-derived macrophages in FcR-signalling. NcDase is significantly increased in the intestinal brush border of the small intestine and induces IgA-bound *Desulfovibrio*, which contributes to up-regulating SCD 1 expression with an increase of MUFAs, further facilitating the development of liver fibrosis. IgG antibodies are also elevated to be against OSEs. This anti-OSE IgG is connected to the differentiation of liver B2 cells to plasma cells. On the contrary, anti-OSE IgM is decreased in the process, suggesting a protective role. Abbreviations: BAFF, B cell-activating factor; HCC, hepatocellular carcinoma; HSCs, hepatic stellate cells; IgA, immunoglobulin-A; IgG, Immunoglobulin-G; IgM, immunoglobulin-M; IFN-γ, interferon gamma; IL-6, interleukin 6; PD-L1, programmed death ligand 1; MyD88, myeloid differentiation primary response 88; MUFAs, monounsaturated fatty acids; NcDase, Neutral ceramidase; MASH, metabolic dysfunction associated steatohepatitis; MASLD-HCC, metabolic dysfunction associated steatotic liver disease-related hepatocellular carcinoma; OSEs, oxidative-stress-derived epitopes; SCD, stearoyl-CoA desaturase; TNF-α, tumor necrosis factor alpha.

B lymphocytes contribute significantly to MASLD-HCC pathogenesis due to their ability to secrete antibodies and various pro- and anti-inflammatory cytokines 131, 132 (Figure 3). B cells heterogeneity is principally defined by surface marker expression, distinguishing B1 and B2 lymphocyte subsets 133, 134. B1 cells are generated from the fetal liver and produce IgM natural antibodies, participating in the innate immune response. Bone marrow-derived B2 precursors differentiate into antibody-secreting plasma cells via Th cell-mediated pathways, producing high-affinity antigen-specific immunoglobulins 131, 135. An additional population of B cells characterized by CD5+CD1d high, known as regulatory B cells (Bregs), produce inhibitory cytokines, such as IL-10, or secrete inhibitory antibodies to affect the function of other immune cells, thus creating an immunosuppressive microenvironment 136, 137.

#### Activation of B cells in MASLD-HCC

Hepatic B cells accumulation occurs alongside an activated, pro-inflammatory phenotype linked to disease severity in MASLD 138-140. MyD88 triggers B cell activation, and its B cell-specific deletion ameliorates inflammation and fibrosis 141. The B cell activation cascade initiates before T cells engagement and features B cell-activating factor (BAFF) overexpression. This cytokine critically sustains B cells survival and developmental progression 138. Using BAFF-neutralizing monoclonal antibodies or BAFF^-/-^ mice can dramatically ameliorate steatohepatitis and reduce liver weight 138, 142. Another potential factor is that gut-derived antigens and bacterial metabolites may drive intrahepatic B cells toward an inflammatory phenotype via MyD88-dependent or BCR signaling pathways. Fecal microbiota transplantation from MASLD patients augments intrahepatic B cells accumulation and hastens disease progression in recipient mice 141. The roles of B cells are discussed from three critical perspectives: antigen presentation, pro-inflammatory cytokine secretion, and the generation of pathogenic antibodies in the progression of MASLD.

#### Antigen presentation

In MASLD, B cells display increased expression of cell surface major histocompatibility complex (MHC)-I and MHC-II, as well as CD86, suggesting enhanced antigen-presenting capability 138, 141. Sometimes intestinal B cells induce T cells hyperactivation that does not rely on their traditional antigen presentation ability but on direct cell-cell interaction via intercellular cell adhesion molecule (ICAM)-1 and leukocyte function-associated antigen (LFA)-1 143. Hepatic B cell-derived cytokines orchestrate pro-inflammatory responses while modulating adjacent T cells activity. Specifically, intrahepatic B cell-secreted IL-6 and TNF-α activate CD4+ T cells and drive their Th1 polarization in MASH pathogenesis 141, 144. B cells also have a prominent role in activating HSCs and promoting fibrosis via TNF signaling 145. Bregs have been shown to promote HCC development, but a subset of Bregs expressing IL-10 has a protective effect against MASH progression 146, 147.

#### Secretion of pro-inflammatory cytokines

Evidence indicates that B cell-derived antibodies are involved in MASLD-HCC pathogenesis. In MASLD patients, elevated serum IgA levels are observed, which activate monocyte-derived macrophages via FcR signaling to promote hepatic fibrosis 143, 148. The IL-21R-STAT1-c-Jun/c-Fos-IgA regulatory pathway is activated during MASLD-HCC, which leads to immunosuppressive IgA+ cells induction 149. These cells can suppress CD8+ T cells by upregulating PD-L1 surface expression and producing the immunosuppressive cytokine IL-10, impacting tumor immune surveillance function in MASLD-HCC 27. MASH models demonstrate elevated neutral ceramidase (NcDase) expression in the small intestinal brush border. NcDase acts as a regulator of gut B cells that induce IgA-bound *Desulfovibrio* and might contribute to up-regulating stearoyl-CoA desaturase (SCD) 1 expression and an increase in monounsaturated fatty acids (MUFAs). The increased SCD1/MUFAs activate Wnt/β-catenin signaling, further facilitating liver fibrosis 150.

#### Generation of pathogenic antibodies

Increased serum IgG2c levels point to a key role for secreted antibodies in MASH pathogenesis. Elevated IgG antibodies against oxidative-stress-derived epitopes (OSEs) have been demonstrated to drive lobular inflammation severity, fibrosis, and increased risk of MASH 138, 151. Plasma cells differentiation from hepatic B2 precursors coincides with rising anti-OSE IgG titers 138, 152. Conversely, there is a significant decline in IgM+B220+ hepatic B cells in MCD mice, paralleled by diminished anti-OSE IgM titers in MASLD patients compared to healthy controls 153. Immunizing low-density lipoprotein receptor knock-out mice (Ldlr^-^/^-^) mice with heat-inactivated pneumococci to induce anti-OxLDL (oxidized low-density lipoproteins) IgM reduces liver inflammation under a high-fat, high-cholesterol diet, highlighting IgM's protective role 154. The potential opposing effects of anti-OSE IgG and IgM demonstrate that the B1 and B2 cells may exert different roles in MASH, creating opportunities for novel therapies targeting specific B cells subsets or antibodies.

In summary, the inflammatory phenotype, pro-inflammatory cytokines, and pathogenic antibodies all play important roles in MASLD development. In other cancer types, substantial evidence supports a correlation between immunotherapy efficacy and B cells infiltration and tertiary lymphoid structure (TLS) formation, which enhance B cell-mediated antitumor immunity 155-157. However, TLS formation may be rare in MASLD-HCC, and this lack might alter B cells function and subsequently impede immunotherapy response. Moreover, given that the generation of antibodies by B cells correlates with response to ICIs in mouse models of triple-negative breast cancer and the abnormal accumulation of antibodies in MASH, these antibodies likely also influence immune responses in MASLD-HCC 158.

## Platelets in MASLD-HCC

While platelets are essential for hemostasis and wound repair, their extended functions now include significant contributions to hepatic inflammation and liver disease pathogenesis 159-161. Hepatic physiology centrally governs platelet biogenesis and elimination. Reciprocally, platelets modulate liver functions through α-granule and dense granule exocytosis, releasing bioactive growth factors and immunoregulatory molecules 162. Typically, patients with MASLD commonly display elevated platelet counts along with increases in mean platelet volume (MPV) and platelet distribution width (PDW) compared to healthy individuals 163, 164. Platelet aggregation is induced by elevated leptin levels, a consequence of adipose tissue accumulation 165, 166. However, a pronounced decrease in platelet counts becomes evident as the disease advances from MASH to hepatic fibrosis. This progressive thrombocytopenia demonstrates utility as a predictor of advancing fibrosis 167. In biopsy-confirmed MASLD cohorts, lower baseline platelet counts correlate with elevated HCC incidence 163. Preclinical studies indicate that simple steatosis and insulin resistance alone fail to elicit increased intrahepatic platelet numbers. This phenomenon manifests only upon progression to MASH, characterized by intrahepatic platelet accumulation, aggregation, and activation 168. Upon activation, platelets shed platelet-derived extracellular vesicles (pEVs) carrying mitochondria with compromised function—evidenced by diminished fatty acid β-oxidation, acetyl-CoA carboxylase 2 (ACC2) inactivation, and defective OXPHOS activity 169. Such mitochondria can transfer to hepatocytes via pEVs, increasing the number of faulty lipid droplet (LD)—bound mitochondria, which disrupts hepatocyte lipid metabolism, causes excess LD buildup, heightened mitochondrial ROS, and apoptosis, and finally aggravates MASH 169. These platelets can also release the α and δ granules laden with myriad molecules including pro-aggregatory molecules such as ADP, serotonin, and thrombin, as well as inflammatory cytokines, chemokines, and growth factors that can directly potentiate inflammatory responses 170, 171. Platelet-derived microparticles (PMPs) also participate in this process 172, 173. Notably, platelet-derived growth factor (PDGF)-β and PDGF-AA drive HSC activation and contribute to biliary fibrosis progression. In contrast, adenosine 5'-triphosphate (ATP) released from platelets suppresses the activation of human HSCs, revealing a complex, multifaceted role for platelets in modulating the fibrotic microenvironment 174-176.

Platelet involvement in MASH progression to HCC exhibits context-dependent complexity, with reports suggesting both pro-tumorigenic and anti-tumor functions. Experimental evidence from CD-HFD mouse models implicates Kupffer cell-mediated platelet recruitment in the liver, facilitated by CD44-hyaluronan binding, as a driver of MASH progression. Critically, platelet-derived glycoprotein Ibα (GPIbα) has been demonstrated as essential for the development of MASH and subsequent HCC in this setting 168. Conversely, studies employing orthotopic implantation of established HCC tumors or carcinogen/oncogene-driven HCC models reveal a protective role for platelets. In these models, platelets upregulate intrahepatic CD8+ T cells accumulation via CD40L release. This platelet-CD40L axis mediates robust anti-tumor immunity in a P2Y12 receptor-dependent manner, thereby inhibiting HCC growth and metastasis 177.

These contradictory findings are likely attributable to distinct experimental models—with the former focusing on platelet involvement in MASLD-driven hepatocarcinogenesis, while the latter specifically examines platelet-mediated modulation of established tumor progression within the MASLD microenvironment. Nevertheless, they converge to suggest that platelets play a pivotal role throughout MASLD pathogenesis.

## Gut Microbiota Modulates Immune Microenvironment, Immunotherapy and MASLD-HCC

The portal vein delivers gut-derived microbial metabolites and microbiota components to the liver, establishing bidirectional gut-liver crosstalk that modulates hepatic physiology 178, 179. Intestinal microbiota regulates liver homeostasis but can also produce damaging molecules and promote pathogenic overgrowth that compromises hepatic integrity 180-182. Germ-free mice are effectively protected from obesity, whereas fecal microbiota transfer from obese mice promotes higher fat accumulation than transfers from lean counterparts 183, 184. Gut microbiota also affects fat storage and fatty liver disease 185. Typically, intestinal epithelial cells sustain gut barrier integrity primarily through tight junction complexes 186, 187. However, during MASLD development and progression, the gut microbiota experiences a decline in diversity, which becomes more significant as the disease advances 188. The abundance of certain microbiota, such as *Streptococcus* and gram-negative microbes, tends to increase 189. These changes can damage the tight junctions between cells, impair gut barrier function, and facilitate portal vein translocation of microbiota and metabolites to the liver 189.

### Translocation of microbial components

Compromised intestinal barrier function enables the pathological transfer of MAMPs, including lipopolysaccharide (LPS) and LTA, to the liver 185. Hepatic TLR4 recognition of gram-negative bacterial LPS initiates NF-κB/MAPK signaling, driving inflammatory cytokine release and potentiating hepatic inflammation 190, 191. The depletion of *Akkermansia muciniphila* (*A. muciniphila*) - a bacterium crucial for intestinal barrier integrity - is observed in fatty liver disease 192, 193. Mechanistically, *A. muciniphila* prevents MASLD-HCC by downregulating γδT cells, upregulating CXCR6+ natural killer T cells (NKT), and inhibiting M1 macrophages polarization through the reduction of hepatic TLR2 expression 192, 194. LTA, as previously indicated, ligation on HSCs orchestrates the activation of ST2-positive Tregs 130.

### Bacterial metabolites

Microbial metabolites, including SCFAs, bile acids, and trimethylamine, may affect the immune system and contribute to MASH and MASLD-HCC 195-197. In MASLD, bacterial metabolites drain into the liver via the portal vein, promoting intrahepatic B cells toward an inflammatory phenotype via MyD88-dependent or BCR signaling, and enhancing antigen presentation and costimulatory molecules expression 141. SCFAs, including butyrate, propionate, and acetate, are enriched in MASLD-HCC patients and drive MASLD progression by regulating hepatic lipogenesis 198-202. Acetate can promote tumor cell proliferation and HCC progression through upregulation of O-GlcNAcylation 181. SCFAs also impact immune cells. Th1 cells exposure to SCFAs activates both signal transducer and activator of transcription 3 (STAT3) and mammalian target of rapamycin (mTOR) pathways, elevating B lymphocyte-induced maturation protein 1 (Blimp-1) expression. This potentiates IL-10 production, mediating anti-inflammatory effects 156. However, some investigators have found that increased circulating SCFA levels protect against inflammation by promoting IL-22 production by CD4+ T cells 157. On the other hand, increased SCFAs can lead to an immunosuppressed response by increasing Tregs and attenuating CD8+ T cells responses 129. Through free fatty acid receptor (FFAR)2 signaling, SCFAs modulate colonic Tregs population dynamics and function while exerting protective effects against colitis 203.

Primary bile acids synthesized in the liver undergo extensive microbial transformation within the intestinal tract. This series of enzymatic reactions—including deconjugation, epimerization, 7-dehydroxylation, reconjugation, 3-acylation, 3-sulfation, and 3-glucosylation—converts them into significant microbiota-derived metabolites that critically influence the progression of MASLD 204. Typically, bile acids act as endocrine mediators that critically maintain glucose and lipid balance via engagement of the G-protein-coupled bile acid receptor 5 (TGR5) and the nuclear farnesoid X receptor (FXR) 205, 206. FXR activation reduces lipid synthesis and glucose levels by modulating SREBP-1C and gluconeogenesis-related genes 207. Similarly, TGR5 helps maintain glucose homeostasis, alleviates hepatic steatosis, and suppresses inflammation 208. Primary bile acids preferentially target FXR, while secondary bile acids prefer to combine with TGR5 209. However, patients with MASH often exhibit elevated total primary bile acids with concurrent reductions in secondary bile acids and 3-indole propionic acid (IPA) 210. During MASLD-HCC, elevated steroidogenic acute regulatory protein 1 (STARD1) expression drives primary bile acid biosynthesis through mitochondrial cholesterol transport. This metabolic reprogramming potentiates cancer stem cell self-renewal, enhances stem-like properties, and amplifies pro-inflammatory signaling in tumor-initiating cells 211. Treatment with anti-cholesterol drugs and manipulation of gut microbiota can completely prevent MASLD-HCC formation 212. Mechanically, the gut microbiome modulates liver CXCL16 expression via bile acids, impacting CXCR6+ NKT cell dynamics. Specifically, primary bile acids boost CXCL16 levels on sinusoidal endothelial cells, whereas secondary bile acids diminish them. This upregulation of CXCL16, the major ligand for CXCR6, drives CXCR6+ NKT cells accumulation in the liver. These accumulated cells are phenotypically activated and secrete elevated IFN-γ upon antigen encounter 213. These findings suggest that primary and secondary bile acids may play distinct roles in MASLD-HCC.

### Changes in intestinal fungi

Studies focusing on intestinal fungi in MASLD mice models are sparse. Previous works demonstrates that mice fed a high-fat diet (HFD) exhibit reduced fungal diversity and constitutional changes 214, 215. This mouse model showed markedly decreased populations of specific fungal taxa, including the genera *Alternaria, Saccharomyces, Septoriella*, and *Tilletiopsis*, along with the species *Saccharomyces cerevisiae* and *Tilletiopsis washingtonensis* 214. Distinct fecal mycobiome profiles distinguish early-stage MASLD patients from advanced-stage counterparts, particularly in non-obese individuals. This dysbiosis correlates with heightened systemic reactivity to* Candida albicans*, evidenced by elevated anti-*C. albicans* IgG titers 216. Furthermore, transferring feces from patients with MASH into a WD-fed gnotobiotic mice model and treating them with antifungal amphotericin B showed reduced liver damage, suggesting that targeting intestinal fungi may be a potential therapy to ameliorate MASH 216.

**Figure 4:**
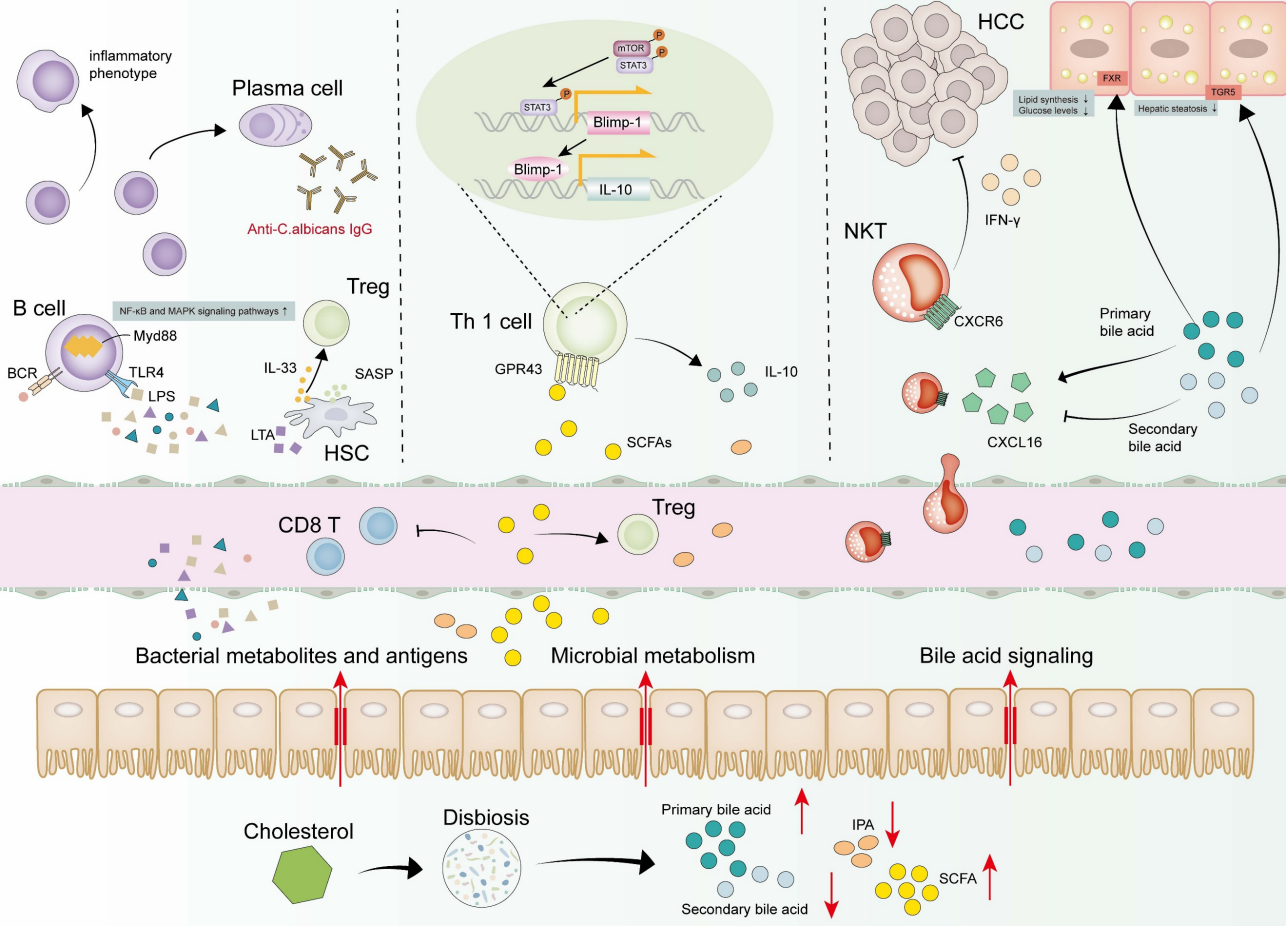
**Dynamic changes of gut microbiota in MASLD-HCC.** Microbial metabolism, bacterial metabolites and antigens are draining into the liver, altering the environment. Bacteria and antigens activate B cells through MyD88-dependent or BCR signaling pathways, which finally induce B cells to differentiate into plasma cells or inflammatory phenotype. LPS binds to TLR4 in the liver and activates the downstream NF-κB and MAPK signaling pathways, leading to the secretion of inflammatory cytokines and promoting liver inflammation. LTA induces a senescent phenotype in HSCs, leading to the release of SASP factors. IL-33 is subsequently exported from senescent HSCs activating Tregs. SCFAs and primary bile acids tend to increase while 3-IPA and secondary bile acids tend to decrease. SCFAs activate STAT3 and mTOR in Th1 cells and upregulate transcription factor Blimp-1 consequently, thus promoting the secretion of IL-10 by Th1 cells. SCFAs also result in an immunosuppressed response by increasing Tregs and attenuating CD8+ T cells responses. Bile acids could bind to the TGR5 and FXR, thereby reducing lipid synthesis and glucose levels. Primary bile acids are used by the gut microbiome to upregulate the expression level of CXCL16 to mediate the accumulation of CXCR6+ NKT cells, which are activated and produce more IFN-γ upon antigen stimulation. Secondary bile acids had a negative effect on CXCL16 expression, causing an opposite result. Abbreviations: Blimp-1, B lymphocyte-induced maturation protein 1; CXCL16, C-X-C motif chemokine ligand 16; CXCR6, C-X-C motif chemokine receptor 6; FXR, farnesoid X receptor; HSC, hepatic stellate cell; IFN-γ, interferon gamma; IL-10, interleukin 10; IL-33, interleukin 33; IPA, indole propionic acid; LPS, lipopolysaccharide; LTA, lipoteichoic acid; mTOR, mammalian target of rapamycin; MyD88, myeloid differentiation primary response 88; MASH, metabolic dysfunction associated steatohepatitis; MASLD-HCC, metabolic dysfunction associated steatotic liver disease-related hepatocellular carcinoma; NKT, natural killer T cells; SCFAs, short-chain fatty acids; STAT3, signal transducer and activator of transcription 3; SASP, senescence-associated secretory phenotype; TGR5, G-protein-coupled bile acid receptor 5; Th, T helper cells; TLR4, Toll-like receptor 4; Tregs, regulatory T cells.

Collectively, gut barrier dysfunction, translocation of microbial components, and dysregulated bacterial metabolite abundance orchestrate MASLD-HCC pathogenesis by affecting the immune system to varying degrees (Figure 4).

## Current Dilemma and Potential Therapeutic Strategies in MASLD-HCC Immunotherapy

**Figure 5:**
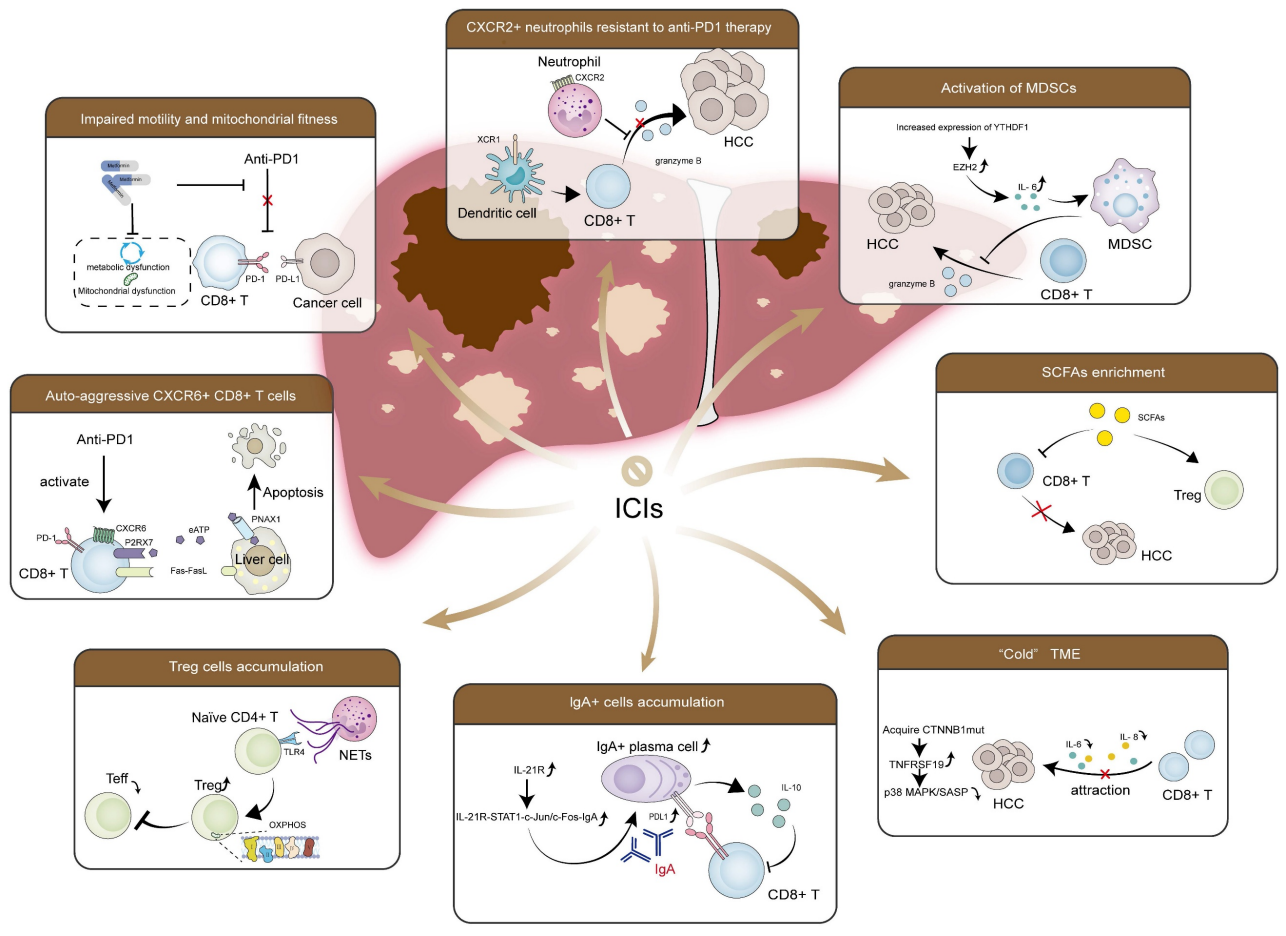
** Key factors contribute to the immunotherapy resistance in MASLD-HCC.** RNA m6A reader protein YTHDF1 is increased, which recruits and activates MDSCs to cause cytotoxic CD8+ T cells dysfunction. CXCR2+ neutrophils secrete pro-tumorigenic cytokines and immunosuppressive molecules, inhibiting the function of CD8+ T cells. The formation of NETs recruits naïve CD4+ T cells and drives their differentiation into Tregs in a TLR4-dependent manner, establishing an immunosuppressive microenvironment. Enriched SCFAs increase Tregs and attenuate CD8+ T cells response, resulting in an immunosuppressed response. IL-21R-STAT1-c-Jun/c-Fos-IgA regulatory pathway is also activated, which leads to the induction of immunosuppressive IgA+ cells. These cells suppress CD8+ T cells by upregulating the expression of PD-L1 on the cell surface and producing the immunosuppressive cytokine IL-10, impairing tumor surveillance function. Accumulated CTNNB1 mutations elevate TNFRSF19 levels, suppressing the secretion of SASP-like cytokines, such as IL-6 and CXCL8, inhibiting the effect of CD8+ T cells. In addition, impaired motility and mitochondrial fitness in CD8+ T cells are observed, along with a low infiltration rate. CXCR6+PD-1+CD8+ T cells are also increased, which causes hepatocyte apoptosis through Fas/FasL interaction, generating an adverse effect in MASLD-HCC immunotherapy. Abbreviations: CXCR, C-X-C motif chemokine receptor; CXCL8, C-X-C motif chemokine ligand 8; CTNNB1, catenin beta 1; Fas, factor associated with suicide; FasL, Fas ligand; HCC, hepatocellular carcinoma; ICIs, immune checkpoint inhibitors; IL-6, interleukin 6; IL-10, interleukin 10; PD-1, programmed death receptor 1; PD-L1, programmed death ligand 1; m6A, N6-methyladenosine; MDSCs, myeloid-derived suppressor cells; MASLD-HCC, metabolic dysfunction associated steatotic liver disease-related hepatocellular carcinoma; NETs, neutrophil extracellular traps; SASP, senescence-associated secretory phenotype; SCFAs, short-chain fatty acids; TNFRSF19, tumor necrosis factor receptor superfamily 19; Tregs, regulatory T cells; YTHDF1, YTH N6-methyladenosine RNA binding protein F1.

Currently, HCC treatment options encompass surgical interventions (resection/transplantation), ablation, intra-arterial therapies, radiotherapy, and systemic therapies. But clinicians choose treatment methods based on disease grading rather than etiology. In MASLD-HCC, excessive lipid accumulation and lipid-induced hepatic insulin resistance, dysregulated metabolism, the gut microbiota and its metabolic products, unique host genetic variants, and chronic inflammation-induced immune response collectively create a complex microenvironment, influencing therapy effectiveness. Current clinical trials focusing on MASLD-HCC remain scarce, most of which have investigated MASLD-HCC as part of non-viral HCC 217. Despite their lack of specificity, these studies' results are still informative. Additionally, meta-analyses are attempting to determine the differences in therapeutic efficacy between MASLD-HCC and other etiologies. As mentioned previously and in former reviews 16, current research suggests that TKIs likely have comparable effectiveness, whereas ICIs might exhibit reduced efficacy in MASLD-HCC compared to viral HCC. Most studies on combination therapy with ICIs also report similar results 217, 218. Mechanically, the distinct TME characteristic of MASLD-HCC, as summarized above-including activation of MDSCs, enriched CXCR2+ neutrophils, Treg cells and IgA+ cells, impaired CD8+ T cells recruitment and effector function, increased specific pro-cancerous CD8+ T cells subsets, accumulated CTNNB1 mutations and elevated SCFAs-may contribute to this phenomenon (Figure 5).

Given the continuum of MASLD progression to HCC, therapeutic interventions targeting early-stage disease may effectively halt hepatocarcinogenesis. Many studies and therapeutic interventions have focused on preventing the progression of MASLD by effectively managing and improving the underlying disease pathology through targeted therapy. Resmetirom currently represents the sole US Food and Drug Administration (FDA)-approved pharmacotherapy for MASLD, exerting therapeutic effects via selective thyroid hormone receptor activation 219. Here, we summarize current therapeutic drugs targeting MASLD and MASLD progression, aiming to reveal the potential strategies for preventing MASLD-HCC (Table 1).

The ongoing elucidation of innate and adaptive immune dynamics within the MASLD-HCC microenvironment provides a mechanistic rationale for modulating these alterations to mitigate hepatocarcinogenesis and enhance immunotherapy efficacy. CXCR2 inhibitors can effectively improve the response of MASLD-HCC to PD-1 therapy by reducing neutrophil infiltration and ROS production 77, 220. Anti-CD122 antibody treatment can decrease CD44+CXCR6+PD-1+CD8+ T cells, thus restoring CD8+ T cells function in MASLD and preventing HCC progression 221. CXCR6+CD8+ T cells activity and function could be modulated therapeutically by targeting IL-15 or FOXO1 94. In the context of MASLD-HCC immunotherapy, metformin enhances CD8+ T cells activity and motility, likely by augmenting mitochondrial mass and promoting mitochondrial activation 94. Furthermore, targeted therapy against activated macrophage subpopulations represents a potential strategy. In murine HCC models, targeting CCR2 effectively **s**uppresses tumor growth and metastasis through limiting TAM infiltration and enhancing CD8+ T cell-mediated antitumor response 222. Besides, targeting the YTHDF1-EZH2-IL-6 signaling axis prevents the recruitment and activation of MDSCs, which could potentially enhance anti-PD-1 efficacy 77. Degradation of NETs by inhibiting their formation or function, e.g., using PAD4 inhibitors or DNase, can help reduce Tregs 223. Employing mitochondria-targeted antioxidants or mitochondrial biogenesis promoters could also help restore the normal metabolic state of CD4+ T cells, thereby reducing their pro-inflammatory effects and preventing tumor progression 224, 225. In addition, targeting the Wnt/β-catenin pathway with ICG001—a small-molecule inhibitor-reversed immune—excluded TME phenotypes in CTNNB1-mutant MASLD-HCC models. This intervention promoted robust CD8+ T cells infiltration and elevated M1/M2 macrophage ratios, indicating restored anti-tumor immunity. Therefore, using ICG001 to reprogram the immune microenvironment toward a pro-inflammatory phenotype may effectively improve the anti-tumor effect of ICIs in MASLD-HCC 101. Targeting IL-21R signaling also has therapeutic potential by reducing the generation of IgA+ cells 149. Since administration of *A. muciniphila* has been shown to decrease body weight, ameliorate IR in obese individuals and restore the efficacy of PD-1-based immunotherapy in cancer patients by increasing CD4+ T cells infiltration in tumors, it is reasonable to speculate that *A. muciniphila* could be used to improve the effect of immune therapy in MASLD-HCC 226, 227. While Tregs promote immunotolerance and thus SCFAs might be considered a potential negative factor in cancer immunotherapy, SCFAs have been shown to enhance the anti-tumor activity of CTLs and the efficacy of CAR T cells in syngeneic murine melanoma and pancreatic cancer models 101. Additional studies are required to elucidate how gut microbiota metabolite-regulated immune microenvironments influence immunotherapy efficacy (Table 2).

Currently, despite some success achieved in preclinical studies targeting specific mechanisms of MASLD-HCC, evaluating specific drugs in clinical trials remains limited. Some drugs are undergoing animal testing. Combining the small molecule CXCR2 inhibitor AZD5069 with anti-PD-1 monoclonal antibody therapy significantly reduces tumor burden and extends survival in a MASLD-HCC mouse model 77. Lipid nanoparticles (LNP)-encapsulated siRNA therapy is an FDA-approved approach for clinical use, and LNP-siRNA or YTHDF1 knockdown in combination with anti-PD-1 therapy has been proven to significantly increase the sensitivity of MASLD-HCC tumors to immunotherapy in mice models 54. Combined metformin and anti-PD-1 therapy also demonstrated good efficacy against MASLD-HCC 94. The efficacy of antiplatelet agents, such as aspirin, and certain antifibrotic drugs has been demonstrated in HCC of other etiologies, including viral-related HCC. However, the therapeutic outcomes of these agents in MASLD-HCC remain inconclusive and warrant further investigation. Consequently, therapeutic development targeting these pathways exhibits significant promise in both preclinical and clinical settings. Prioritizing investigation of the aforementioned targets and agents represents a strategic approach to enhance immunotherapy efficacy and advance curative strategies for MASLD-HCC.

## Conclusion and Future Perspectives

MASLD critically drives HCC development by progressively remodeling the hepatic immune microenvironment. This remodeling occurs throughout the disease spectrum, from steatosis to steatohepatitis, fibrosis, and ultimately HCC, where shifts in the metabolic landscape alter immune cell phenotypes/function and gut microbial communities. These changes, in turn, influence MASLD progression, HCC development, and immunotherapy efficacy. The metabolic alterations in MASLD are multifaceted, extending beyond the widely recognized dysregulated lipid metabolism to encompass pivotal alterations in ammonia and glutamine handling 228. Clinical evidence indicates elevated systemic ammonia levels and progressive downregulation of glutamine synthetase in MASH patients compared to simple steatosis 228. Notably, enhanced glutamine catabolism—a hallmark metabolic adaptation in cancer—manifests in HCC through overexpression of glutaminase 1 (GLS1), which catalyzes **t**he conversion of glutamine to glutamate. GLS1 inhibition attenuates tumor proliferation and suppresses epithelial-mesenchymal transition (EMT) 229. Critically, GLS1 upregulation correlates with advanced clinicopathological features and stemness phenotypes, mechanistically driven by ROS-mediated activation of Wnt/β-catenin signaling that sustains cancer stemness 230. Parallel investigations reveal that sustained hyperammonemia promotes HSC activation and fibrogenesis in MASLD models. Aberrant GLS1 induction exacerbates oxidative stress, impairs very-low-density lipoprotein (VLDL) particle assembly, and ultimately potentiates hepatocyte lipid accumulation and MASH progression 231. Novel diagnostic strategies employing dynamic monitoring of glutamine flux through GLS expression patterns establish its potential as a noninvasive biomarker for detecting hepatic malignancies 232.

This review synthesizes alterations in immune cell dynamics and gut microbiota composition during MASH and MASLD-HCC pathogenesis. Notably, mechanistic insights into the MASH-HCC transition and immunotherapy response patterns in this patient population remain inadequately explored. First, existing MASLD models in mice cannot effectively mimic the pathophysiological signature of human MASLD. Second, consistent conclusions are difficult to obtain using the numerous different rodent experimental models of MASLD and various HCC models in the MASH context (Table 3). Comparative analysis of MASLD rodent models is complicated by their differential recapitulation of human disease pathophysiology 233. Furthermore, not all cases of MASH progress to liver tumors, and many studies on MASH and MASLD do not adequately address pathogenesis and treatment of MASLD-HCC. This lack of focus limits our understanding of the immune microenvironment and the treatment options for MASLD-HCC. Therefore, it is essential to conduct more studies and develop more relevant animal models for MASH progression to HCC in the future.

The response rate to immunotherapy is poor in MASLD-HCC. ICIs therapy seems to have encountered a significant setback in treating MASLD-HCC patients. Activation of MDSCs, enriched CXCR2+ neutrophils, Tregs, and IgA+ cells, diminished CD8+ T cells recruitment and functional impairment, increased specific pro-cancerous CD8+ T cells subsets, accumulated CTNNB1 mutations and elevated SCFAs may collectively contribute to this phenomenon. Research on gut dysbiosis-driven MASLD pathogenesis has elucidated key mechanisms centered on intestinal barrier compromise, which permits hepatic translocation of microbial components and metabolites (e.g., SCFAs, bile acids). These hepatotropic signals orchestrate immunometabolic reprogramming via upregulation of CXCR6+ NKT cells, suppressing M1 macrophages polarization, expanding Tregs, and attenuating CD8+ T cells effector functions. Compelling preclinical evidence demonstrates that microbiota—directed interventions-including fecal microbiota transplantation, probiotics, prebiotics, and synthetic biotics—effectively restore enteric homeostasis and ameliorate metabolic dysregulation and inflammation in MASLD models.

Advances in multi-omics sequencing have propelled tumor precision medicine into clinical focus, utilizing molecular and genetic profiling to tailor cancer therapies based on individual tumor characteristics. Methodologically, integrative analysis of tumor transcriptomes and patient prognoses has yielded the SAHR (Score of Aggregated Hazard Ratio) model—a universal quantitative metric for assessing clinical aggressiveness. Applying this framework to HCC revealed three molecular subtypes with distinct prognostic outcomes among Asian populations, each exhibiting multifaceted molecular disparities 234. Notably in HBV-related HCC, proteogenomic profiling of 159 patients through integrated multi-omics analysis (encompassing somatic mutations, copy number alterations, transcriptomics, proteomics, and phosphoproteomics) delineated three tumor subclusters with characteristic pathway activation patterns. This approach further identified pyrroline-5-carboxylate reductase 2 (PYCR2) and alcohol dehydrogenase 1A (ADH1A) as robust prognostic biomarkers, with mechanistic studies confirming their roles in modulating pro-tumorigenic metabolic pathways 235. However, such comprehensive multi-omics research on MASLD-HCC remains scarce. Spatial mapping at single-cell resolution reveals a previously unappreciated heterogeneity in immune cell distribution within MASLD-HCC. Contrary to prior models, immune cells are most abundant in adjacent non-tumor tissue, diminishing progressively towards the tumor core. Furthermore, spatial interactions shift from T cell networks towards immunosuppressive connections involving MDSCs and TAMs, potentially disrupting antitumor immunity 100. Hence, further exploration of this heterogeneity in the distribution and composition of immune cells is warranted and may provide new directions for understanding immunotherapy resistance in MASLD-HCC. Every new mechanistic discovery may become the key to unlocking potential solutions to complex problems.

Elucidating dynamic alterations in immune cell populations and gut microbiota during MASLD-HCC progression not only enhances our understanding of the disease process but also provides a foundation for solving therapeutic dilemma and identifying new drugs and targets. Nowadays, with the underlying reason for immunotherapy resistance and immunometabolic changes in MASLD-HCC gradually being revealed, new targets have already been floated. However, specific drugs undergoing evaluation in clinical trials and validated in robust animal models are still limited. The lack of etiology-based classification of treatments also contributes to the scarcity of targeted research on MASLD-HCC. Future studies must focus on addressing existing research gaps in these areas, exploring additional reasons for immunotherapy resistance, identifying novel targets, and developing effective drugs to achieve a cure for MASLD-HCC ultimately. With the rising prevalence of MASLD-HCC continuing unabated, clarifying the mechanisms linking the metabolic microenvironment and immune responses in MASLD-HCC will be crucial not only to enhance treatment efficacy but also to minimize side effects and enable personalized treatment for patients with this condition.

## Figures and Tables

**Table 1:** Current therapy drugs and treatment targeting MASLD progression

Treatment	Mechanism	Drug name	Other interventions	Study phase	Clinical trial number or reference	Results
**PPAR (PPARα/PPARγ/PPARδ) agonist**	Promote the polarization of macrophages towards M2 type and reduce inflammatory response	Rosiglitazone	NA	II	NCT00492700,^236^	Insulin sensitivity and ALT levels are elevated, liver fat deposition is reduced
		Pioglitazone	NA	IV	NCT00227110	Liver fat deposition and fibrosis are reduced
		Pioglitazone	Metformin	IV	NCT03796975	Liver fat deposition and fibrosis are reduced
		Pioglitazone	Empagliflozin	IV	NCT03646292	Ongoing
		Pioglitazone	Dapagliflozin	NA	NCT06649162	Ongoing
		GW501516	NA	Preclinical	237, 238	Liver fat deposition and fibrosis are reduced
		Lanifibranor	NA	III	NCT04849728	Liver fibrosis is reduced
		Lanifibranor	Empagliflozin	II	NCT05232071	Ongoing
		Elafibranor	NA	II	NCT01694849	Liver fibrosis is reduced
**CCR2/CCR5 antagonist**	Block the CCR5/CCR2 and reduce hepatic infiltration of monocytes/macrophages	Cenicriviroc	NA	II	NCT02217475	Liver fibrosis is reduced
		Cenicriviroc	Tropifexor	II	NCT03517540	Liver fat deposition and fibrosis are reduced
		Maraviroc	NA	Preclinical	239	Liver fat deposition and fibrosis are reduced
**CXCR2 antagonist**	Block CXCL1-CXCR2 axis and inhibit neutrophils and macrophages recruitment	RS10289	NA	Preclinical	240	Inflammatory responses, liver steatosis and liver damage are reduced
		RS504393	NA	Preclinical	241	Inflammatory responses, liver steatosis and liver damage are reduced
**THR-β agonist**	Promote fatty acid oxidation and reduce liver fat accumulation by activating THR-β	Resmetirom	NA	III	NCT05500222	Liver fat deposition and fibrosis are reduced
		ASC41	NA	I	NCT04686994	The drug was well-tolerated and no safety concerns were found
		TERN-501	NA	II	NCT05415722	Liver fat deposition and fibrosis are reduced
		TERN-501	TERN-101	II	NCT05415722	Liver fat deposition and fibrosis are reduced
		HSK31679	NA	II	NCT06168383	Ongoing
		VK2809	NA	II	NCT02927184	Cholesterol and liver fibrosis are reduced
		Kylo-0603	NA	I	NCT06365580	Ongoing
		ECC4703	NA	I	NCT05552274	Ongoing
		ALG-055009	NA	I	NCT05090111	Ongoing
**IL-6 receptor antagonist**	Inhibit IL-6-mediated signalling and attenuate inflammatory response and injury in hepatocytes by blocking the IL-6 receptor	Tocilizumab	NA	Preclinical	242	Liver fat deposition and fibrosis are reduced
**NE inhibitor**	Reduce hepatic inflammation and early hepatic fibrosis by inhibiting NE activity and ameliorate early inflammation in MASLD-HCC	Sivelestat	NA	Preclinical	71, 223	Insulin sensitivity is improved, inflammatory responses, liver steatosis and liver damage are reduced
**Angiotensin II receptor antagonist**	Inhibit TGF-β signalling pathway and reduce hepatic fibrosis, thereby ameliorating hepatic infiltration of CD8+ T cells	Losartan	NA	II	NCT00699036	Ongoing
**mTOR inhibitor**	Regulate Th17/Treg cells balance and enhance the immunosuppressive function of Treg cells, thereby attenuating MASH-associated inflammation and fibrosis	Rapamycin	NA	Preclinical	243	Liver fat deposition and fibrosis are reduced
		AZD2014	NA	Preclinical	244, 245	Liver fat deposition is reduced, insulin sensitivity is improved, but there are some side effects (hyperglycemia and hyperlipidaemia)
		Everolimus	NA	Preclinical	246	Adipose synthesis and inflammatory responses are reduced
**Antiplatelet drug**	Inhibit platelet activation and aggregation by irreversibly inhibiting cyclooxygenase (COX-1) and reduce thromboxane A2 (TXA2) production	Aspirin	NA	I/II	NCT04031729	Liver fat deposition is reduced
	A phosphodiesterase inhibitor that works by inhibiting platelet aggregation and promoting blood vessel dilation	Cilostazol	NA	I/II	NCT04761848	Ongoing
	Inhibit P2Y12 receptor on platelets and block ADP-mediated platelet activation	Clopidogrel	NA	Preclinical	247	Platelet activation and hepatocyte inflammation are reduced
**FXR/TGR5 agonist**	Activate FXR, regulate the synthesis, secretion and reabsorption of bile acids, which helps to reduce liver fat accumulation	Obeticholic Acid	NA	II	NCT01265498	Liver fibrosis is reduced
		Obeticholic Acid	Atorvastatin	II	NCT02633956	LDLc and liver fibrosis are reduced
		Cilofexor	NA	II	NCT03987074	Liver fibrosis is reduced
		Cilofexor	Semaglutide& Firsocostat	II	NCT03987074	Liver fibrosis is reduced
		EDP-305	NA	II	NCT03421431, 248	ALT level is elevated, and liver fat deposition is reduced
		Tropifexor	NA	Preclinical	249	ALT level is elevated, and liver fat deposition is reduced
		Tropifexor	Cenicriviroc	II	NCT03517540	Liver fat deposition and fibrosis are reduced
		MET409	NA	II	NCT04702490	Liver fat deposition is reduced
		TERN-101	NA	II	NCT04328077	Liver fat deposition is reduced
		TERN-101	TERN-501	II	NCT05415722	Liver fat deposition and fibrosis are reduced
	Activate TGR5, regulate the synthesis, secretion and reabsorption of bile acids, which helps to reduce liver fat accumulation	INT-777	NA	Preclinical	250	Inflammatory responses, liver steatosis and liver damage are reduced
	FXR/TGR5 double agonist	INT-767	NA	Preclinical	250	Inflammatory responses, liver steatosis and liver damage are reduced
**Probiotics/prebiotics/synbiotics**	Regulate intestinal flora, reduce the abundance of harmful bacteria, strengthen intestinal barrier function, and reduce inflammatory response	Lactobacillus	Bifidobacterium	NA	NCT03467282,^251^	Ongoing, but former study has shown reduced ALT and AST levels and improved liver steatosis
		Bifidobacterium	Lactobacillus	NA	NCT03467282,^252^	Ongoing
		VSL#3	NA	I/II	NCT03511365	Obvious benefit was not observed
		Oligofructose	NA	NA	NCT02568605	Liver fibrosis is reduced
		Oligofructose-enriched inuli	NA	NA	NCT03184376	Liver fibrosis is reduced
**GLP-1 agonist/GLP-1 receptor agonist**	Activate PI3K/Akt signalling pathway and inhibit NF-κB signalling pathway, improve insulin sensitivity and prevent hepatocytes apoptosis	Liraglutide	NA	II	NCT01237119	ALT level is elevated, and liver fat deposition is reduced
		Tirzepatide	NA	II	NCT04166773	Liver fibrosis is reduced
		Retatrutide	NA		253	Liver fat deposition is reduced
		Exenatide	NA	II/III	NCT00650546	Liver fat deposition is reduced
		Efinopegdutide	NA	I	NCT06052566	Ongoing
		Semaglutide	NA	II	NCT02970942	Liver fibrosis is reduced
		Semaglutide	Cilofexor	II	NCT04971785	Liver fibrosis is reduced
		Semaglutide	Firsocostat	II	NCT04971785	Liver fibrosis is reduced
		Semaglutide	Firsocostat&Cilofexor	II	NCT03987074	Liver fibrosis is reduced
		Semaglutide	NNC0194-0499	I	NCT05766709	Ongoing
		Cotadutide	NA	II	NCT04019561	Liver fat deposition is reduced
		HM15211	NA	I	NCT03744182	The drug was well-tolerated and no safety concerns were found
**ACC inhibitor**	Inhibit acetyl coenzyme A carboxylase (ACC) and reduce fat synthesis	Firsocostat	NA	II	NCT02856555	Liver fat deposition is reduced
		Firsocostat	Semaglutide	II	NCT04971785	Liver fat deposition and fibrosis are reduced
		Firsocostat	Semaglutide&Cilofexor	II	NCT03987074	Liver fat deposition and fibrosis are reduced
		PF-05221304	NA	II	NCT03248882	Liver fat deposition is reduced
		PF-05221304	PF-06865571	II	NCT03776175	Liver fat deposition is reduced
		WZ66	NA	Preclinical	254	Liver fat deposition is reduced
**FASN inhibitors**	Inhibit fat synthase (FASN) and reduce Th17 cells differentiation, fat synthesis and pro-inflammatory effects	Denifanstat (TVB-2640)	NA	III	NCT04906421	Liver fibrosis is reduced
		TVB-3664	NA	Preclinical	255	Triglyceride levels and liver fat are decreased
		FT-4101	NA	Preclinical	255	Liver fat deposition and fibrosis are reduced
**Caspase inhibitor**	Inhibit the activity of caspase enzyme, reduce apoptosis and inflammation	Emricasan	NA	II	NCT02686762	Fail to improve liver histology in patients with MASH fibrosis despite target engagement and may have worsened fibrosis and ballooning
		GS-9450	NA	II	NCT00740610	Prevents apoptosis
		VX-166	NA	Preclinical	256	Rate of apoptosis and inflammatory factor levels in hepatocytes are reduced
**Gal-3 inhibitors**	Inhibit galactoglucan-3 (Gal-3) and reduce inflammation and fibrosis	Belapectin (GR-MD-02)	NA	II/III	NCT04365868	Liver histology is improved, and oesophageal varices are prevented
		GM-CT-01	NA	Preclinical	257	Inflammatory responses and liver fibrosis are reduced
		GB1211	NA	I	NCT03809052	The drug was well tolerated and no safety concerns were found
**LOXL2 inhibitor**	Inhibit lysyl oxidase-like protein-2 (LOXL2) and reduce collagen cross-linking and fibrosis	Simtuzumab	NA	II	NCT01672866	Failed to achieve the desired effect
		Solithromycin	NA	II	NCT02510599	Liver fibrosis is reduced
**ASK1 inhibitor**	Inhibit apoptosis signaling regulation kinase 1 (ASK1) and reduce apoptosis and fibrosis	Selonsertib	NA	III	NCT03053050	Liver fibrosis is reduced
		SRT-015	NA	I	NCT04887038	Ongoing
**DGAT2 inhibitor**	Inhibit diacylglycerol acyltransferase 2 (DGAT2) and reduce triacylglycerol incorporation in the liver	ION224	NA	II	NCT04932512	Liver fat deposition and fibrosis are reduced

**Table 2:** Potential therapeutic targets/strategies addressing the mechanisms of immunotherapy resistance in MASLD-HCC

Potential therapeutic targets/strategy	Mechanism	Cancer type	Reference	Specific Drugs
**Akkermansia muciniphila**	Increase the infiltration of CD4+ T cells	MASLD-HCC	226, 227	NA
**Anti-CD122 antibody**	Decrease the amount of CD44+CXCR6+PD-1+CD8+ T cells	MASLD-HCC	221	NA
**CCR2 inhibitor**	Reduce the infiltration of TAMs and reinvigorate the antitumor activity of CD8+ T cells	HCC	222	NA
**CXCR2 inhibitor**	Reducing CXCR2+ neutrophils infiltration and ROS production	MASLD-HCC	77, 220	AZD5069
**SCFAs**	Decrease the amount of SCFAs to reduce Tregs and improve CD8+ T cells responses	MASLD-HCC	NA	NA
**IL-21R signalling blockade**	Decrease the amount of IgA+ cells and improve CD8+ T cells responses	MASLD-HCC	149	NA
**Modulate IL-15 or FOXO1**	Alter the activity and function of CXCR6+CD8+ T cells	MASLD-HCC	94	NA
**PAD4 inhibitor/Dnase**	Degradate of NETs by inhibiting their formation or function and reducing Tregs	MASLD-HCC	223	NA
**CTNNB1 mutation**	Inhibit Wnt/β-catenin pathway and reprogram the immune microenvironment towards a pro-inflammatory phenotype	HCC	100	ICG001
**YTHDF1-EZH2-IL-6 signaling axis**	Decrease the expression of YTHDF and recruitment of MDSCs	MASLD-HCC	54	LNP-siRNA
**Metformin**	Increase mitochondrial mass and activation of CD8+ T cells	MASLD-HCC	94	Metformin

**Table 3:** Roles of different immune cells in different MASLD-HCC models

Cell subset	Mouse Models	Mechanism	Reference
**Macrophages**	DEN + HFD	MyD88 in myoblasts enhances MASLD-HCC development by promoting M2 macrophages polarization	52
	Myeloid-Lineage-Specific Heterozygous Deletion of Ncoa5 mice	NCOA5 deficiency in macrophages as a key factor in the transition of MASH to HCC	22, 53
	DEN + CDA-HFD	The loss of NRG4 induces TAM-like macrophages and exhausted cytotoxic CD8+ T cells in MASLD-HCC	21
**Neutrophils**	Streptozotocin + HFD	NETs regulate the OXPHOS of naïve CD4+ T cells, drive their differentiation into Tregs	24, 60
	Tumor cells + WD or DEN + ALIOS	Pro-tumorigenic cytokines and immunosuppressive molecules secreted by CXCR2+ neutrophils	77
**Dendritic cells**	Tumor cells + WD or DEN + ALIOS	XCR1+ cDC1 mediate cDC1 and CD8 T cells interactions	77
**CD8+ cytotoxic T cells**	Spatial transcriptomics	The infiltration is diminished within tumor regions	100
	CD-HFD	Induce liver damage, upregulate exhausted markers and promoting HCC, auto-aggressive killing hepatocytes	19, 94, 96
	DEN + CD-HFD or DEN + HFHC	YTHDF1 suppresses cytotoxic CD8+ T cells function by enhancing the secretion of IL-6	54
	Spatial transcriptomics	CD8+PD-1+ T cells inducible T cells ICOS+, MDSCs, and tumor-TAMs	100
	MCD/CDAA/WD	Reduce cell motility, impaire metabolic fitness	25, 94
**B cells**	HFD-fed MUP-uPA mice	IgA+ cells suppress CD8+ T cells, produce the immunosuppressive cytokine IL-10	27
**Platelets**	CD-HFD	Recruit of CD8+ T cells and NKT cells, drive HCC	168
	Tumor cells + MCD or DEN + CDAA or CCl4 + WD	Upregulate the accumulation of CD8+ T cells, inhibiting the growth and metastasis of HCC in MASH	177

## Abbreviations

ACC2: acetyl-CoA carboxylase 2
ADAM17: a disintegrin and metalloproteinase 17
ADH1A: alcohol dehydrogenase 1A
APOE: apolipoprotein E
ATP: adenosine 5'-triphosphate
BAFF: B cell-activating factor
Blimp-1: B lymphocyte-induced maturation protein 1
Bregs: regulatory B cells
CCL2: C-C motif chemokine ligand 2
CCR2: C-C motif chemokine receptor 2
CDAA: choline-deficient and amino acid-defined diet
cDCs: conventional dendritic cells
cDC1: conventional type I dendritic cells
cDC2: conventional type II dendritic cells
CD-HFD: choline-deficient, high-fat diet
CPT: carnitine palmitoyltransferase
CELA1: chymotrypsin-like elastase family member 1
CTLA4: anti-cytotoxic T-lymphocyte antigen 4
CTNNB1: catenin beta 1
CXCL10: C-X-C motif chemokine ligand 10
CX3CR1: C-X3-C motif chemokine receptor 1
CXCR6: C-X-C motif chemokine receptor 6
DCs: dendritic cells
EmKC: embryo-derived Kupffer cells
EMT: epithelial-mesenchymal transition
Fas: factor associated with suicide
FasL: Fas ligand
FFAR: free fatty acid receptor
FOXO1: Forkhead Box O1
FXR: farnesoid X receptor
GPIbα: glycoprotein Ibα
GZMB: granzyme B
GSDMD: gasdermin D
HBV: hepatitis B virus
HCC: hepatocellular carcinoma
HCV: hepatitis C virus
HFD: high-fat diet
HIF: hypoxia-inducible factor
HNPs: human neutrophil peptides
HSCs: hepatic stellate cells
ihTh17: inflammatory hepatic CXCR3+ Th17
ICAM: intercellular cell adhesion molecule
ICIs: immune checkpoint inhibitors
ICOS: inducible T cell costimulator
IFN-γ: interferon gamma
IgA: immunoglobulin-A
IgG: immunoglobulin-G
IgM: immunoglobulin-M
IR: insulin resistance
IL-1β: interleukin 1 beta
IPA: indole propionic acid
LCs: Langerhans cells
LCN: lipocalin
LD: lipid droplet
LDLR: low density lipoprotein receptor
LFA: leukocyte function-associated antigen
LNP: lipid nanoparticles
LPS: lipopolysaccharide
LTA: lipoteichoic acid
mTOR: mammalian target of rapamycin
m6A: N6-methyladenosine
MAdCAM-1: mucosal addressin cell adhesion molecule-1
MASH: metabolic dysfunction-associated steatohepatitis
MASLD: metabolic dysfunction-associated steatotic liver disease
MCP-1: monocyte chemoattractant protein-1
MCD: methionine-choline-deficient diet
MDSCs: myeloid-derived suppressor cells
MHC: major histocompatibility complex
MPO: myeloperoxidase
MPV: mean platelet volumes
MR: magnetic resonance
MT: metallothionein
MUFAs: monounsaturated fatty acids
MyD88: myeloid differentiation primary response 88
NAFLD: non-alcoholic fatty liver disease
MASLD-HCC: metabolic dysfunction-associated steatotic liver disease-related hepatocellular carcinoma
MAMPs: microbial-associated molecular patterns
NcDase: neutral ceramidase
NCOA5: nuclear receptor coactivator 5
NKT: natural killer T cells
NRG4: neuregulin 4
NE: neutrophil elastase
NETs: neutrophil extracellular traps
OSE: oxidative-stress-derived epitopes
OxLDL: oxidized low-density lipoproteins
OXPHOS: oxidative phosphorylation
pDCs: plasmacytoid dendritic cells
pEVs: platelet-derived extracellular vesicles
PD-1: programmed death receptor-1
PD-L1: programmed death-ligand 1
PUFAs: polyunsaturated fatty acids
PRRs: pattern recognition receptors
PDGF: platelet-derived growth factor
PDW: platelet distribution width
PMPs: platelet-derived microparticles
PYCR2: pyrroline-5-carboxylate reductase 2
ROS: reactive oxygen species
SASP: senescence-associated secretory phenotype
SAHR: Score of Aggregated Hazard Ratio
SCD: stearoyl-CoA desaturase
SCFAs: short-chain fatty acids
STARD1: steroidogenic acute regulatory protein 1
STAT3: signal transducer and activator of transcription 3
TAMs: tumor-associated macrophages
TG: triglyceride
TGF-β: transforming growth factor beta
TGR5: G-protein-coupled bile acid receptor 5
Th: T helper
Tregs: regulatory T cells
TREM2: triggering receptor expressed on myeloid cells 2
Trm: tissue-resident memory T cells
TKIs: tyrosine kinase inhibitors
TLRs: Toll-like receptors
TLS: tertiary lymphoid structures
TME: tumor microenvironment
TNF: tumor necrosis factor
TNFRSF19: tumor necrosis factor receptor superfamily 19
XCR1: X-C receptor 1
YTHDF1: YT521-B homology (YTH) m6A RNA-binding protein 1 (YTHDF1)
WD: western diet
VLDL: very-low-density lipoprotein
